# Supplementary material for: Tissue-Specific 1H-NMR Metabolomic Profiling in Mice with Adenine-Induced Chronic Kidney Disease
Source: Metabolites. 2021 Jan 10;11(1):45. doi: 10.3390/metabo11010045 (PMC7827497; doi:10.3390/metabo11010045)
Supplement: Supplementary file 1 [file metabolites-11-00045-s001.pdf]

metabolites

Article

Tissue-specific ^{1}H-NMR metabolomic profiling in mice with adenine-induced chronic kidney disease

Ram B. Khattri [1], Trace Thome [1], and Terence E. Ryan [1,2,3,*]

[1] Department of Applied Physiology and Kinesiology, University of Florida, Gainesville, FL, 32611; USA
[2] Center for Exercise Science, University of Florida, Gainesville, FL, 32611; USA
[3] Myology Institute, University of Florida, Gainesville, FL, 32611; USA

* Correspondence: Terence E. Ryan, Ph.D., Email: ryant@ufl.edu; Tel.: +01-352-294-1700 (T.E.R.)

Received: date; Accepted: date; Published: date

Supplemental Data

Below are supplement figures and tables related to the above referenced manuscript.

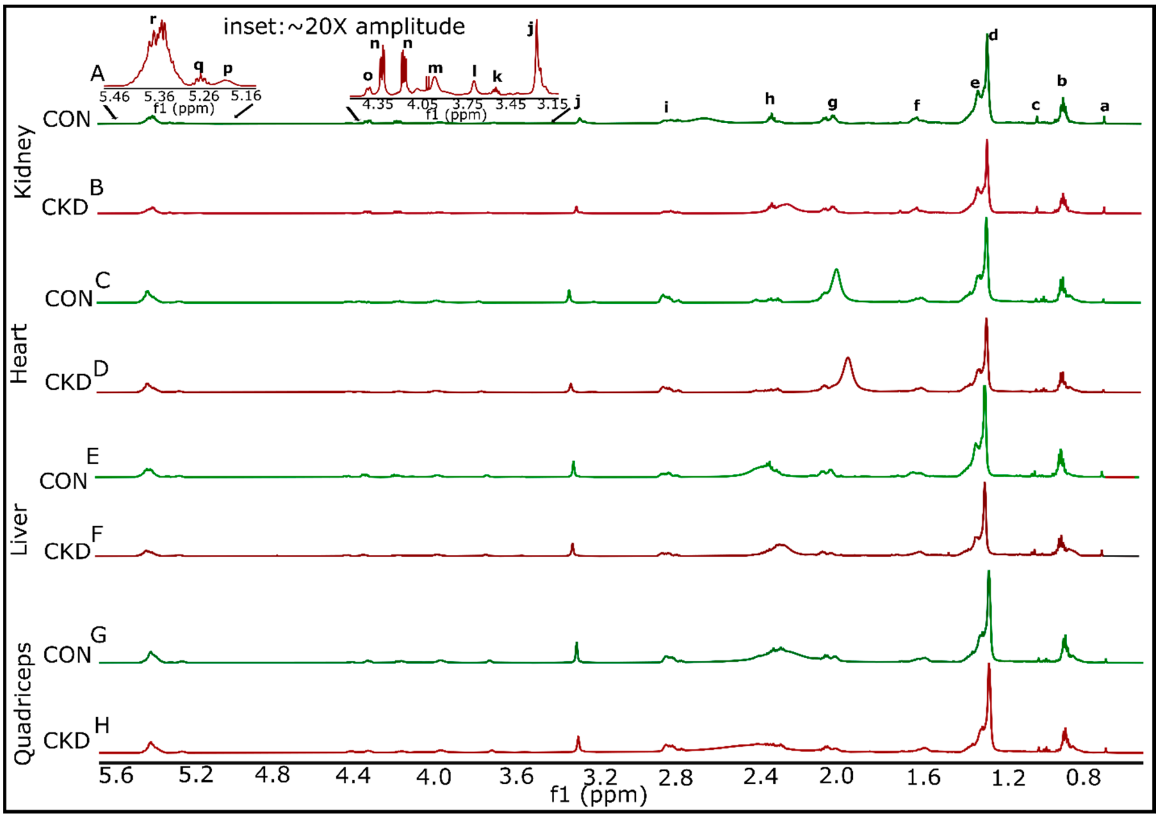

Figure S1: Representative ^{1}H NMR spectra for the organic phase samples from both control and CKD groups. Figure 2 A & B are Kidney control#5 (12.3 mg) and CKD#5 (12.5 mg), respectively; C & D are heart control#5 (6.8 mg) and CKD#5 (6.8 mg), respectively, E & F are liver control#4 (13.2 mg) and CKD#4 (12.0 mg), respectively and G & H are quadriceps control#6 (14.7 mg) and CKD#6 (15.2 mg), respectively. 'a' is (CH_3) cholesterol/cholesterol ester (C18) , 'b' is (CH_3) cholesterol/esterified and free fatty acids, 'c' is (CH_3) cholesterol (C19), 'd' is cholesterol, 'e' is (CH_2)$_n$ of aliphatic chains, 'f' is (**CH**$_2$-CH$_2$-COO-) β-methylene protons associated to carbon groups, 'g' is (**CH**$_2$-CH=CH-CH$_2$) α-methylene protons associated to double bonds, 'h' is (**CH**$_2$-CCO-) α-methylene protons associated to carbonyl groups, 'I' is (=CH-**CH**$_2$-CH=CH)divinyl methylene protons of w-3 and w-6 unsaturated fatty acids, 'j' is N^+(**CH**$_3$)$_3$ in phosphatidylcholine, choline & sphingomyelin, 'k' is cholesterol, 'l' is glycerophospholipids, 'm' is (3**CH**$_2$-)glycerophospholipids, 'n' is (-**CH**$_2$-) triglyceride, 'o' is (1CH-) phospholipid and triglyceride, 'p' is (CH-) phospholipid, 'q' is (**CH**-)triglyceride, and 'r' is (-**CH**=**CH**-) protons in double bonds in unsaturated fatty acids and –**CH** from cholesterol.

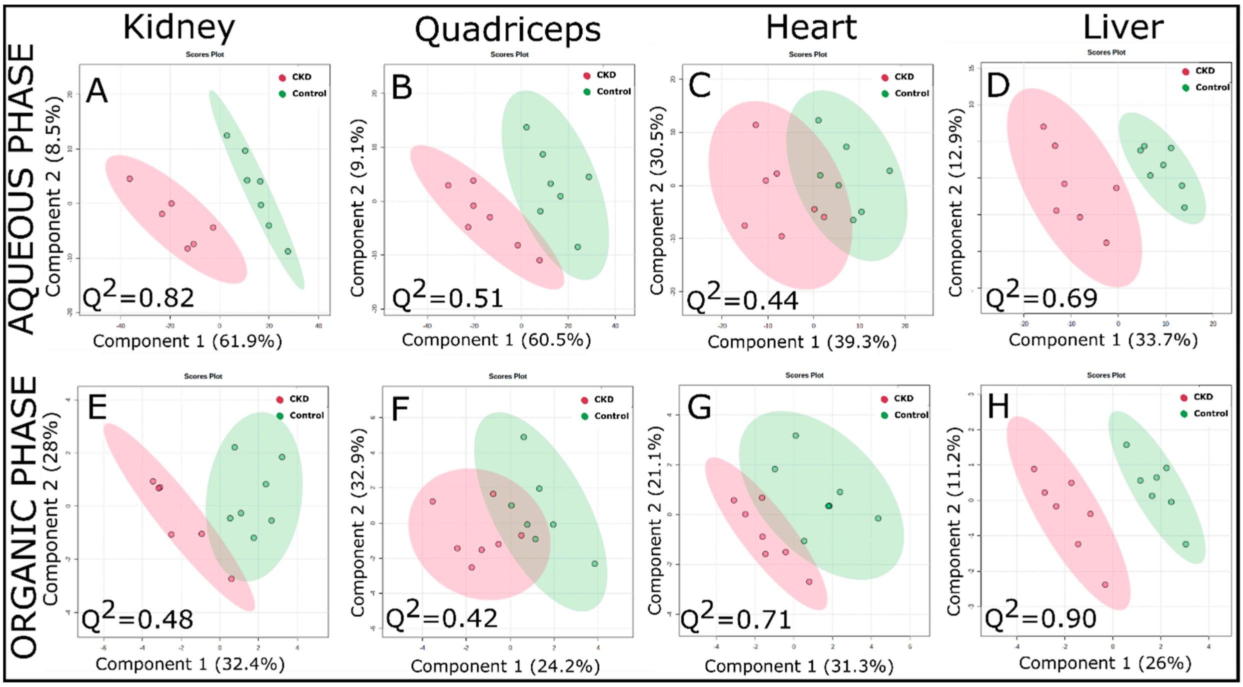

Figure S2. PLS-DA score plots obtained from the ¹H-NMR spectra of extracted tissue samples (using targeted profiling approach) from control and adenine-induced CKD groups. Figure S2 A, B, C, & D are the PLS-DA score plots from the aqueous phase samples for kidney, heart, liver, and quadriceps, respectively. Similarly, Figure S2 E, F, G, & H are the PLS-DA score plots from the organic phase samples from the above tissue samples maintaining same order. (n=7/group/tissue).

Table S1. Table showing average concentration (mM) ± S.D. of Metabolites (for aqueous phase samples) and average peak areas (A.U.) ± S.D. for peaks/lipid compounds (for organic phase samples) along with their respective p-value (from two-tailed unpaired Student's *t*-test)

**Kidney: Aqueous phase via ^{1}H NMR**				
S.No.	Metabolites	Average concentration (mM) ± S.D.		p-value from unpaired t-test
		Control	CKD	Control vs CKD
1	Leucine	0.127 ± 0.021	0.078 ± 0.021	0.0026
2	Valine	0.109 ± 0.022	0.069 ± 0.019	0.0077
3	Isoleucine	0.067 ± 0.014	0.040 ± 0.012	0.0057
4	Isobutyrate	0.041 ± 0.011	0.026 ± 0.008	0.0246
5	Lactate	3.069 ± 0.505	2.121 ± 0.850	0.0432
6	Alanine	0.069 ± 0.015	0.044 ± 0.016	0.0218
7	Lysine	0.380 ± 0.116	0.264 ± 0.088	n.s.
8	Acetate	0.071 ± 0.022	0.058 ± 0.025	n.s.
9	Citrate	0.040 ± 0.026	0.244 ± 0.097	0.0005
10	Glutamate	1.346 ± 0.200	0.488 ± 0.285	0.0003
11	Pyruvate	0.062 ± 0.018	0.051 ± 0.018	n.s.
12	Succinate	0.216 ± 0.032	0.056 ± 0.036	<0.0001
13	Myo-inositol	0.776 ± 0.146	0.616 ± 0.220	n.s.
14	Glutamine	0.201 ± 0.047	0.090 ± 0.031	0.0008
15	Aspartate	0.288 ± 0.007	0.076 ± 0.062	0.0004
16	Creatine	0.215 ± 0.090	1.141 ± 0.035	n.s.
17	Creatinine	0.030 ± 0.008	0.024 ± 0.004	n.s.
18	O-phosphocholine	0.215 ± 0.039	0.156 ± 0.077	n.s.
19	Sn-Glycero-3-Phosphocholine	0.729 ± 0.199	0.482 ± 0.138	0.0381
20	Trimethyl-N-oxide	0.691 ± 0.240	0.208 ± 0.208	0.0047
21	Taurine	2.223 ± 0.362	1.904 ± 0.395	n.s.

22	Glycine	0.736 ± 0.111	0.205 ± 0.112	<0.0001
23	ATP/AMP	0.133 ± 0.023	0.046 ± 0.035	0.0005
24	Inosine	0.147 ± 0.071	0.079 ± 0.067	n.s.
25	α-Glucose	0.252 ± 0.110	0.381 ± 0.075	0.0471
26	NAD^+	0.050 ± 0.013	0.006 ± 0.004	<0.0001
27	Allantoin	0.011 ± 0.006	0.013 ± 0.006	n.s.
28	Sucrose	0.007 ± 0.010	0.018 ± 0.006	n.s.
29	Uracil	0.028 ± 0.014	0.023 ± 0.006	n.s.
30	Uridine Triphosphate	0.050 ± 0.022	0.021 ± 0.011	0.0221
31	Fumarate	0.023 ± 0.008	0.009 ± 0.003	0.0028
32	Tyrosine	0.056 ± 0.018	0.022 ± 0.007	0.0020
33	Histidine	0.029 ± 0.003	0.013 ± 0.007	0.0003
34	Tryptophan	0.055 ± 0.007	0.015 ± 0.005	<0.0001
35	Phenylalanine	0.074 ± 0.017	0.015 ± 0.005	n.s.
36	Benzoate	0.085 ± 0.033	0.138 ± 0.057	n.s.
37	Nicotinurate	0.080 ± 0.010	0.043 ± 0.015	0.0006
38	NADH	0.010 ± 0.002	0.003 ± 0.003	0.0009
39	Formate	0.045 ± 0.025	0.048 ± 0.024	n.s.
**Kidney: Organic phase via ^{1}H NMR**				
	Peak/Lipid component	Average peak intensity (A.U.) ± S.D.		p-value from unpaired t-test
		Control	CKD	Control vs CKD
1	(CH_3) cholesterol/cholesterol ester (C18)	19.40 ± 2.12	15.81 ± 2.06	0.0162
2	(CH_3) cholesterol/esterified and free fatty acids	356.61± 58.92	244.92 ± 45.39	0.0052
3	(CH_3) cholesterol (C19)	21.70 ± 2.00	17.71 ± 2.26	0.0099
4	$(CH_2)_n$ of Cholesterol	683.03 ± 123.47	396.94 ± 103.48	0.0017

5	$(CH_2)_n$ of aliphatic chains	766.84 ± 224.11	470.63 ± 167.45	0.0323
6	(C**H**$_2$-CH$_2$-COO-) β-methylene protons associated to carbon groups	146.20 ± 32.17	103.59 ± 23.36	0.0323
7	(C**H**$_2$-CH=CH-CH$_2$) α-methylene protons associated to double bonds	202.33 ± 52.53	130.36 ± 70.28	n.s.
8	(C**H**$_2$-CCO-) α-methylene protons associated to carbonyl groups	96.65 ± 32.25	49.89 ± 56.60	n.s.
9	(=CH-C**H**$_2$-CH=CH)divinyl methylene protons of w-3 and w-6 unsaturated fatty acids	164.03 ± 52.64	52.85 ± 15.68	0.0008
10	N^+(C**H**$_3$)$_3$ in phosphadidylcholine, choline & sphingomyelin	59.35 ± 12.50	40.18 ± 8.64	0.0142
11	(3C**H**$_2$-)glycerophospholipids	42.05 ± 7.86	22.46 ± 6.44	0.0009
12	(-C**H**$_2$-) triglyceride1	24.71 ± 9.08	13.32 ± 7.39	0.0454
13	(-C**H**$_2$-) triglyceride2	29.24± 11.24	15.52 ± 8.71	0.0471
14	1CH-Phospholipids + triglyceride	13.60 ± 2.87	7.36 ± 2.94	0.0046
15	CH-Phospholipid	15.27 ± 2.67	7.33 ± 2.66	0.0005
16	(C**H**-)triglyceride	8.10 ± 5.26	4.21 ± 3.26	n.s.
17	is (-C**H**=C**H**-) protons in double bonds in unsaturated fatty acids and –C**H** from cholesterol	202.38 ± 34.82	97.80 ± 32.05	0.0003
**Heart: Aqueous phase via ^{1}H NMR**				
S.No.	Metabolites	Average concentration (mM) ± S.D.		p-value from unpaired t-test
		Control	CKD	Control vs CKD

1	Leucine	0.064 ± 0.016	0.041 ± 0.010	0.0104
2	Valine	0.049 ± 0.012	0.031 ± 0.007	0.0085
3	Isoleucine	0.029 ± 0.008	0.018 ± 0.004	0.0137
4	Isobutyrate	0.007 ± 0.002	0.005 ± 0.001	n.s.
5	Lactate	2.069 ± 0.466	2.216 ± 0.615	n.s.
6	Alanine	0.396 ± 0.097	0.298 ± 0.069	n.s.
7	Lysine	0.294 ± 0.081	0.204 ± 0.066	n.s.
8	Acetate	0.132 ± 0.054	0.088 ± 0.037	n.s.
9	Citrate	0.044 ± 0.009	0.033 ± 0.010	n.s.
10	Glutamate	0.669 ± 0.118	0.470 ± 0.150	0.0278
11	Pyruvate	0.150 ± 0.061	0.090 ± 0.027	0.0507
12	Succinate	0.135 ± 0.016	0.115 ± 0.037	n.s.
13	Glutamine	0.788 ± 0.188	0.572 ± 0.162	n.s.
14	Aspartate	0.128 ± 0.053	0.071 ± 0.036	n.s.
15	N,N-Dimethylformamide	0.020 ± 0.006	0.018 ± 0.008	n.s.
16	Creatine	1.497 ± 0.239	1.173 ± 0.327	n.s.
17	Creatinine	0.114 ± 0.031	0.092 ± 0.030	n.s.
18	O-phosphocholine	0.085 ± 0.028	0.072 ± 0.028	n.s.
19	Sn-Glycero-3-Phosphocholine	0.152 ± 0.041	0.103 ± 0.038	n.s.
20	Trimethyl-N-oxide	0.951 ± 0.372	0.882 ± 0.279	n.s.
21	Taurine	3.770 ± 0.602	3.057 ± 0.841	n.s.
22	Glycine	0.072 ± 0.015	0.065 ± 0.017	n.s.
23	ATP/AMP	0.196 ± 0.038	0.164 ± 0.043	n.s.
24	Inosine	0.184 ± 0.038	0.147 ± 0.038	n.s.
25	α-Glucose	0.033 ± 0.014	0.060 ± 0.027	n.s.
26	NAD^+	0.042 ± 0.004	0.033 ± 0.012	n.s.
27	Fumarate	0.018 ± 0.006	0.014 ± 0.004	n.s.

28	Tyrosine	0.025 ± 0.007	0.009 ± 0.004	0.0003
29	Histidine	0.031 ± 0.004	0.019 ± 0.006	0.0025
30	Tryptophan	0.097 ± 0.045	0.054 ± 0.018	0.0511
31	Phenylalanine	0.044 ± 0.015	0.028 ± 0.008	0.0340
32	Benzoate	0.102 ± 0.039	0.079 ± 0.031	n.s.
33	Nicotinurate	0.046 ± 0.016	0.034 ± 0.007	n.s.
34	Formate	0.096 ± 0.045	0.057 ± 0.018	n.s.
**Heart: Organic phase via ^{1}H NMR**				
S.No.	Peak/Lipid component	Average peak intensity (A.U.) ± S.D.		p-value from unpaired t-test
		Control	CKD	Control vs CKD
1	(CH_3) cholesterol/cholesterol ester (C18)	4.14 ± 0.74	3.06 ± 0.71	0.0238
2	(CH_3) cholesterol/esterified and free fatty acids	212.16 ± 38.66	172.44 ± 50.88	n.s.
3	(CH_3) cholesterol (C19)	6.83 ± 1.31	5.38 ± 1.37	n.s.
4	$(CH_2)_n$ of Cholesterol	413.48 ± 46.35	315.91 ± 80.25	0.0241
5	$(CH_2)_n$ of aliphatic chains	279.09 ± 47.81	215.47 ± 55.20	n.s.
6	(C**H**$_2$-CH_2-COO-) β-methylene protons associated to carbon groups	64.78 ± 11.01	49.92 ± 12.52	0.0495
7	(C**H**$_2$-CH=CH-CH_2) α-methylene protons associated to double bonds	67.91 ± 31.39	48.41 ± 16.62	n.s.
8	(C**H**$_2$-CCO-) α-methylene protons associated to carbonyl groups	82.93 ± 6.85	59.95 ± 17.93	0.0125
9	(=CH-C**H**$_2$-CH=CH)divinyl methylene protons of w-3 and w-6 unsaturated fatty acids	73.49 ± 13.43	53.26 ± 11.76	0.0150

10	$N^+(CH_3)_3$ in phosphadidylcholine, choline & sphingomyelin	38.78 ± 5.91	28.61 ± 7.79	0.0255
11	(3CH_2-)glycerophospholipids	21.49 ± 3.41	17.16 ± 4.20	n.s.
12	(-CH_2-) triglyceride1	13.54 ± 1.80	8.43 ± 1.97	0.0005
13	(-CH_2-) triglyceride2	4.84± 2.15	2.14 ± 1.07	0.0175
14	1CH-Phospholipids + triglyceride	9.58 ± 2.17	8.46 ± 1.94	n.s.
15	CH-Phospholipid	9.49 ± 1.48	7.61 ± 1.81	n.s.
16	(CH-)triglyceride	1.73 ± 0.45	0.65 ± 0.29	0.0003
17	is (-CH=CH-) protons in double bonds in unsaturated fatty acids and –CH from cholesterol	108.79 ± 31.47	85.49 ± 18.81	n.s.
**Liver: Aqueous phase via ^{1}H NMR**				
S.No.	Metabolites	Average concentration (mM) ± S.D.		p-value from unpaired t-test
		Control	CKD	Control vs CKD
1	Leucine	0.237 ± 0.055	0.178 ± 0.041	0.0583
2	Valine	0.196 ± 0.048	0.143 ± 0.038	0.0584
3	Isoleucine	0.108± 0.026	0.087 ± 0.024	n.s.
4	Isobutyrate	0.072 ± 0.015	0.066 ± 0.024	n.s.
5	Lactate	5.812 ± 0.399	4.034 ± 0.849	0.0006
6	Alanine	2.684 ± 0.740	2.198 ± 0.657	n.s.
7	Lysine	0.538 ± 0.096	0.405 ± 0.082	0.0240
8	Acetate	0.239 ± 0.035	0.221 ± 0.064	n.s.
9	Citrate	0.040 ± 0.012	0.047 ± 0.017	n.s.
10	Glutamate	0.944 ± 0.207	0.676 ± 0.154	0.0255
11	2-Aminoadipate	0.690 ± 0.321	0.397 ± 0.105	n.s.

12	Pyruvate	0.094 ± 0.018	0.091 ± 0.035	n.s.
13	Succinate	0.366 ± 0.063	0.289 ± 0.063	n.s.
14	Glutamine	0.669 ± 0.185	0.603 ± 0.120	n.s.
15	Aspartate	0.150 ± 0.067	0.135 ± 0.044	n.s.
16	Glutathione	0.856 ± 0.085	0.570 ± 0.123	0.005
17	Creatine	0.115 ± 0.017	0.100 ± 0.023	n.s.
18	Creatinine	0.045 ± 0.007	0.037 ± 0.008	n.s.
19	Taurine	0.3.467 ± 1.530	5.717 ± 1.430	0.0219
20	α-Glucose	8.551 ± 1.110	8.484 ± 1.795	n.s.
21	Mannose	0.022 ± 0.015	0.037 ± 0.019	n.s.
22	Glycine	0.427 ± 0.051	0.457 ± 0.074	n.s.
23	UDP-N-Acetylglucosamine	0.024 ± 0.017	0.055 ± 0.033	n.s.
24	ATP/AMP	0.191 ± 0.025	0.180 ± 0.040	n.s.
25	Uridine Triphosphate	0.090 ± 0.040	0.086 ± 0.047	n.s.
26	Inosine	0.029 ± 0.015	0.050 ± 0.016	0.0319
27	NAD^+	0.021 ± 0.004	0.017 ± 0.009	n.s.
28	Fumarate	0.011 ± 0.003	0.021 ± 0.007	0.0041
29	Tyrosine	0.054 ± 0.016	0.033 ± 0.008	0.0129
30	Histidine	0.098 ± 0.023	0.100 ± 0.027	n.s.
31	Tryptophan	0.050 ± 0.013	0.059 ± 0.027	n.s.
32	Phenylalanine	0.081 ± 0.015	0.069 ± 0.015	n.s.
33	Benzoate	0.073 ± 0.019	0.076 ± 0.039	n.s.
34	Nicotinurate	0.113 ± 0.018	0.125 ± 0.024	n.s.
35	Formate	0.015 ± 0.006	0.035 ± 0.019	0.0309
**Liver: Organic phase via ^{1}H NMR**				
S.No.	Peak/Lipid component	Average peak intensity (A.U.) ± S.D.		p-value from unpaired t-test

		Control	CKD	Control vs CKD
1	(CH_3) cholesterol/cholesterol ester (C18)	13.09 ± 1.47	12.34 ± 1.53	n.s.
2	(CH_3) cholesterol/esterified and free fatty acids	378.67 ± 60.81	339.55 ± 73.46	n.s.
3	(CH_3) cholesterol (C19)	15.15 ± 2.28	15.30 ± 2.39	n.s.
4	$(CH_2)_n$ of Cholesterol	626.69 ± 61.56	471.30 ± 72.80	0.0018
5	$(CH_2)_n$ of aliphatic chains	983.32 ± 138.18	627.15 ± 120.98	0.0005
6	(**CH**$_2$-CH_2-COO-) β-methylene protons associated to carbon groups	159.52 ± 20.65	113.99 ± 18.15	0.0016
7	(**CH**$_2$-CH=CH-CH_2) α-methylene protons associated to double bonds	202.48 ± 51.14	142.40 ± 64.57	n.s.
8	(**CH**$_2$-CCO-) α-methylene protons associated to carbonyl groups	240.61 ± 88.98	179.92 ± 90.74	n.s.
9	(=CH-C**H**$_2$-CH=CH)divinyl methylene protons of w-3 and w-6 unsaturated fatty acids	170.28 ± 87.13	95.49 ± 8.58	n.s.
10	N^+(**CH**$_3$)$_3$ in phosphadidylcholine, choline & sphingomyelin	84.48 ± 16.87	60.83 ± 21.24	n.s.
11	(3C**H**$_2$-)glycerophospholipids	36.71 ± 4.05	26.74 ± 6.71	0.0089
12	(-**CH**$_2$-) triglyceride1	30.56 ± 5.01	14.33 ± 2.99	<0.0001
13	(-**CH**$_2$-) triglyceride2	32.22 ± 5.73	15.49 ± 3.05	<0.0001
14	1CH-Phospholipids + triglyceride	17.25 ± 1.84	12.73 ± 3.03	0.0086
15	CH-Phospholipid	18.76 ± 2.26	14.41 ± 2.51	0.0090
16	(**CH**-)triglyceride	11.03 ± 2.66	3.62 ± 1.69	<0.0001
17	is (-**CH**=**CH**-) protons in double bonds in unsaturated	216.71 ± 29.42	146.59 ± 17.23	0.0003

	fatty acids and –CH from cholesterol			
**Quadriceps: Aqueous phase via ^{1}H NMR**				
S.No.	Metabolites	Average concentration (mM) ± S.D.		p-value from unpaired t-test
		Control	CKD	Control vs CKD
1	Leucine	0.101 ± 0.026	0.075 ± 0.024	n.s.
2	Valine	0.135 ± 0.028	0.092 ± 0.029	0.0231
3	Isoleucine	0.056 ± 0.014	0.040 ± 0.013	0.0586
4	Isobutyrate	0.010 ± 0.003	0.009 ± 0.003	n.s.
5	Lactate	9.247 ± 1.259	7.512 ± 1.908	n.s.
6	Alanine	1.022 ± 0.177	0.661 ± 0.169	0.0035
7	Lysine	0.401 ± 0.090	0.384 ± 0.195	n.s.
8	Acetate	0.130 ± 0.069	0.089 ± 0.030	n.s.
9	Citrate	0.082 ± 0.029	0.068 ± 0.011	n.s.
10	Glutamate	0.593 ± 0.137	0.292 ± 0.133	0.0023
11	Pyruvate	0.092 ± 0.040	0.065 ± 0.026	n.s.
12	Succinate	0.090 ± 0.034	0.055 ± 0.029	n.s.
13	Glutamine	0.746 ± 0.228	0.627 ± 0.137	n.s.
14	Aspartate	0.092 ± 0.034	0.083 ± 0.022	n.s.
15	Trimethylamine	0.015 ± 0.001	0.014 ± 0.004	n.s.
16	Creatine	9.136 ± 0.747	8.820 ± 2.209	n.s.
17	Creatinine	0.638 ± 0.131	0.592 ± 0.208	n.s.
18	Glycerol	0.097 ± 0.018	0.128 ± 0.034	n.s.
19	Anserine	3.647 ± 0.279	3.676 ± 0.845	n.s.
20	Trimethyl-N-oxide	0.990 ± 0.220	0.710 ± 0.324	n.s.
21	Taurine	13.883 ± 1.542	14.697 ± 0.679	n.s.
22	Glycine	0.467 ± 0.129	0.378 ± 0.131	n.s.

23	Creatine phosphate	0.684 ± 0.179	0.622 ± 0.230	n.s.
24	ATP/AMP	0.648 ± 0.201	0.530 ± 0.158	n.s.
25	α-Glucose	0.387 ± 0.083	0.455 ± 0.081	n.s.
26	NAD^+	0.088 ± 0.019	0.070 ± 0.014	n.s.
27	Fumarate	0.021 ± 0.008	0.012 ± 0.004	0.0381
28	Tyrosine	0.091 ± 0.030	0.036 ± 0.013	0.0013
29	Histidine	0.644 ± 0.115	0.404 ± 0.121	0.0042
30	Tryptophan	0.081 ± 0.037	0.059 ± 0.021	n.s.
31	Phenylalanine	0.093 ± 0.030	0.071 ± 0.022	n.s.
32	Benzoate	0.107 ± 0.028	0.074 ± 0.029	n.s.
33	Nicotinurate	0.039 ± 0.009	0.032 ± 0.009	n.s.
34	Formate	0.092 ± 0.040	0.060 ± 0.027	n.s.
**Quadriceps: Organic phase via ^{1}H NMR**				
S.No.	Peak/Lipid component	Average peak intensity (A.U.) ± S.D.		p-value from unpaired t-test
		Control	CKD	Control vs CKD
1	(CH_3) cholesterol/cholesterol ester (C18)	6.45 ± 0.91	6.28 ± 0.89	n.s.
2	(CH_3) cholesterol/esterified and free fatty acids	268.37 ± 58.40	209.28 ± 39.76	n.s.
3	(CH_3) cholesterol (C19)	9.87 ± 1.49	9.14 ± 1.23	n.s.
4	$(CH_2)_n$ of Cholesterol	651.12 ± 130.00	524.04 ± 114.43	n.s.
5	$(CH_2)_n$ of aliphatic chains	439.08 ± 128.46	323.61 ± 120.13	n.s.
6	(**CH_2**-CH_2-COO-) β-methylene protons associated to carbon groups	104.94 ± 27.65	85.19 ± 26.56	n.s.
7	(**CH_2**-CH=CH-CH_2) α-methylene protons associated to double bonds	127.98 ± 38.01	95.09 ± 32.34	n.s.

8	(CH_2-CCO-) α-methylene protons associated to carbonyl groups	177.75 ± 111.02	137.37 ± 58.81	n.s.
9	(=CH-CH_2-CH=CH)divinyl methylene protons of w-3 and w-6 unsaturated fatty acids	174.66 ± 84.06	117.82 ± 41.07	n.s.
10	$N^+(CH_3)_3$ in phosphadidylcholine, choline & sphingomyelin	101.29 ± 18.05	76.17 ± 9.97	0.0175
11	(3CH_2-)glycerophospholipids	43.49 ± 7.15	30.89 ± 5.56	0.0082
12	(-CH_2-) triglyceride1	29.34 ± 6.68	20.72 ± 8.30	n.s.
13	(-CH_2-) triglyceride2	30.04 ± 8.72	23.96 ± 8.84	n.s.
14	1CH-Phospholipids + triglyceride	19.77 ± 6.51	16.35 ± 5.68	n.s.
15	CH-Phospholipid	18.07 ± 3.09	13.76 ± 1.83	0.0187
16	(**CH**-)triglyceride	4.14 ± 2.62	2.47 ± 2.46	n.s.
17	is (-**CH**=**CH**-) protons in double bonds in unsaturated fatty acids and –**CH** from cholesterol	177.37 ± 33.98	138.49 ± 29.16	n.s.

Table S2. Metabolites that were responsible for driving separation among the two groups for all aqueous phase tissue samples (obtained from VIP-score plots of PLS-DA analysis) are shown. Here, 's' means singlet, 'd' represents doublet, 'dd' is for doublet of doublet, and 'm' stands for multiplet.

Spectra range (ppm)	Metabolite	Peak Pattern	VIP scores			
			Kidney	Heart	Liver	Quad
3.24-3.27	Taurine	t	~3.5	~2.2	N.A.	~4.5
3.23-3.24	Trimethyl-N-oxide	s	~2.8	~4	N.A.	~0.3
1.31-1.33	Lactate	d	~2.3	~4.3	~4.0	~0.2
3.51-3.54	Myo-inositol	dd	~1.9	N.A.	N.A.	N.A.
3.22-3.23	Sn-Glycero-3-Phosphocholine	s	~1.7	~0.4	N.A.	N.A.
2.32-2.36	Glutamate	m	~1.6	N.A.	~1.5	~1.1
3.55	Glycine	s	~1.5	~0.6	~1.1	N.A.
2.51-2.54	Citrate	d	~1.5	N.A.	N.A.	N.A.
2.39-2.40	Succinate	s	~1.3	N.A.	N.A.	N.A.
3.21	O-phosphocholine	s	~1.2	~0.5	N.A.	N.A.
3.02-3.03	Creatine	s	N.A.	~1.0	N.A.	~3.2
3.75-3.77	Anserine	s	N.A.	N.A.	N.A.	~2.0
1.46-1.48	Alanine	d	N.A.	N.A.	N.A.	~1.1
3.03-3.04	Creatinine + PCr	s	N.A.	N.A.	N.A.	~0.6
3.53-3.55	Glycerol	d	N.A.	N.A.	N.A.	~0.2
0.94-0.97	Leucine	t	N.A.	~0.2	~0.5	N.A.
0.88-0.89	Pantothenate	s	N.A.	~0.2	N.A.	N.A.
3.51-3.54	Glucose	dd	N.A.	N.A.	~1.6	N.A.
2.94-2.99	Glutathione	dd	N.A.	N.A.	~2.1	N.A.
6.12-6.14	ATP/AMP	d	N.A.	N.A.	~1.4	N.A.
2.22-2.25	2-Aminoadipate	s	N.A.	N.A.	~1.4	N.A.
1.69-1.76	Lysine	m	N.A.	N.A.	~0.7	N.A.

Table S3. Peaks/lipid compounds that were responsible for driving separation among the two groups for all organic phase tissue samples (obtained from VIP-score plots of PLS-DA analysis) are shown. Here, 's' means singlet, 'd' represents doublet, 'dd' is for doublet of doublet, and 'm' stands for multiplet.

Spectra range (ppm)	Lipid class	Associated protons	Peak Pattern	VIP scores			
				Kidney	Heart	Liver	Quad
5.29-5.42	Fatty acids	CH=CH	m	~1.8	~0.7	~1.0	~1.5
1.54-1.64	Phospholipid/Triglyceride	(C**H**$_2$-CH$_2$-COO)	m	~1.5	~0.6	~0.3	N.A.
0.69-0.66/1.0-1.01	Cholesterol	C18/C19 C**H**$_3$	s	~1.5	~1.0	~1.5	~1.4
2.75-2.86	Fatty acids	HC=CH-C**H**$_2$-HC=CH	m	~1.4	~0.3	~0.7	~1.2
0.78-0.92	Fatty acids/cholesterol/phospholipids	C**H**$_3$	m	~1.4	~1.0	~1.0	0.2
5.18-5.23	Phospholipid	(C**H**-)	m	~1.4	~1.0	~0.4	~1.3
3.32-3.40	Phosphatidylcholine	N(C**H**$_3$)$_3^+$	s	~0.9	N.A.	~0.2	~0.6
3.89-3.97	Glycerophospholipids	(3C**H**$_2$-)	m	~0.7	~0.6	N.A.	~1.3
2.22-2.33	Triglyceride /Phopholipids	(C**H**$_2$-CCO-)	m	~0.6	~0.4	N.A.	~0.1
4.26-4.32	Triglyceride	C**H**$_2$	dd	~0.4	~1.8	~1.9	~1.6
1.26-1.39	Fatty acids /Triglyceride /Phopholipids	(-C**H**$_2$)$_n$	m	~0.3	~0.7	~1.1	~1.3
5.24-5.28	Triglyceride	(C**H**-)	m	~0.2	~2.0	~1.8	N.A.
1.19-1.27	Cholesterol	(-C**H**$_2$)$_n$	Broad s	N.A.	N.A.	~0.3	~0.7
4.34-4.42	Triglyceride /Phopholipids	(1C**H**-)	m	N.A.	~1.6	N.A.	~0.2
2.03-2.07	Fatty acids /Triglyceride /Phopholipids	(-C**H**$_2$-HC=CH-CH$_2$)	m	N.A.	0.2	~0.1	N.A.

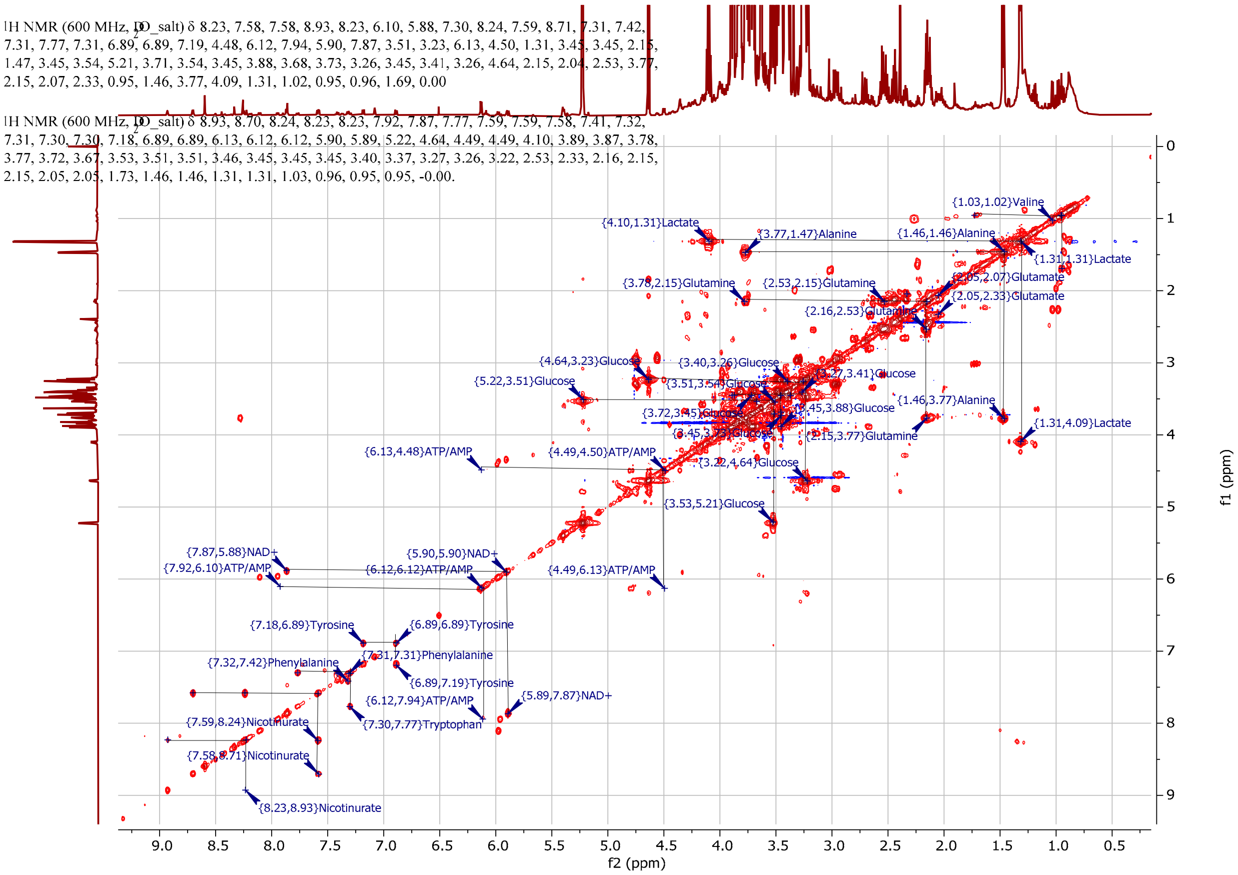

Figure S3. A fraction of COSY spectrum for the liver sample (CKD 1 liver - aqueous phase) showing few metabolites present in slightly higher concentration.

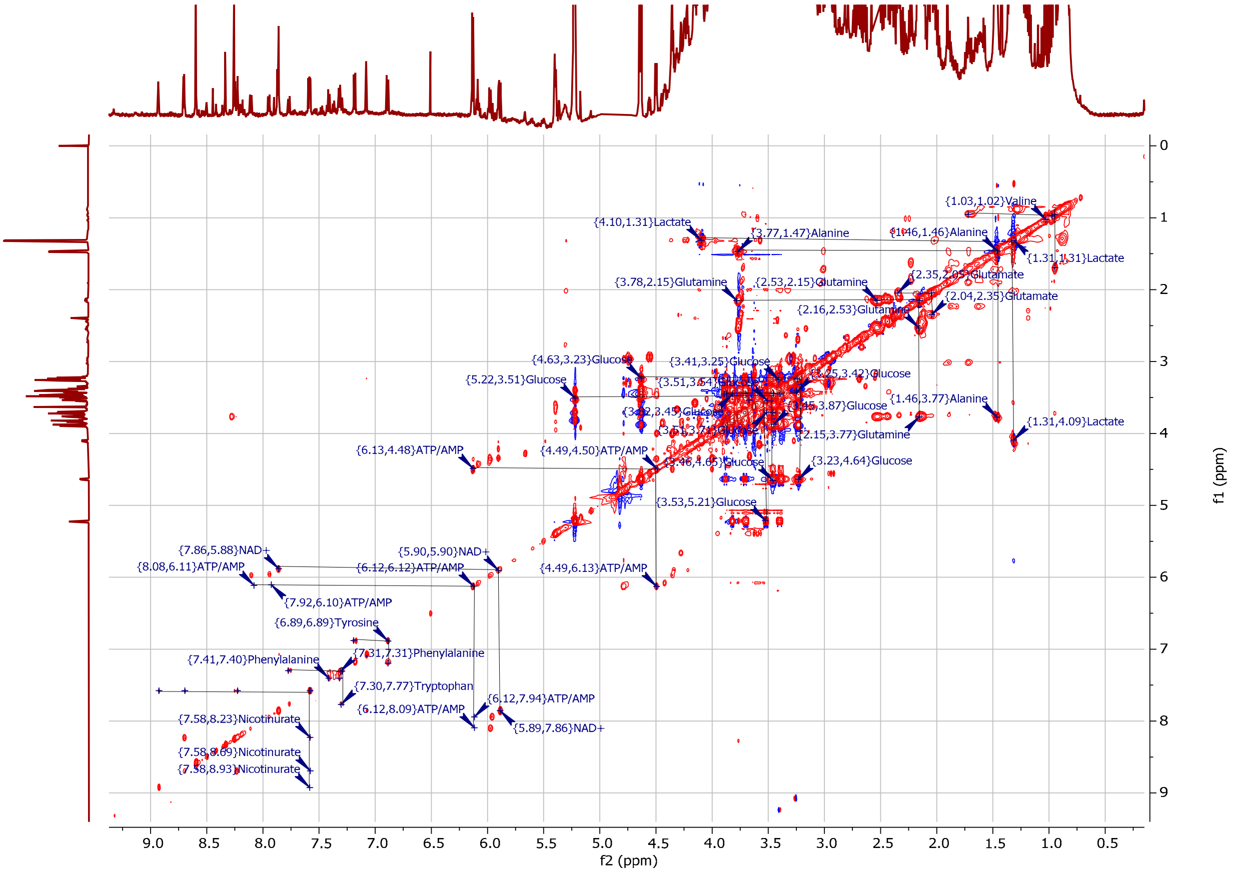

Figure S4. A fraction of TOCSY spectrum for the liver sample (CKD 1 liver - aqueous phase) showing few metabolites present in slightly higher concentration.

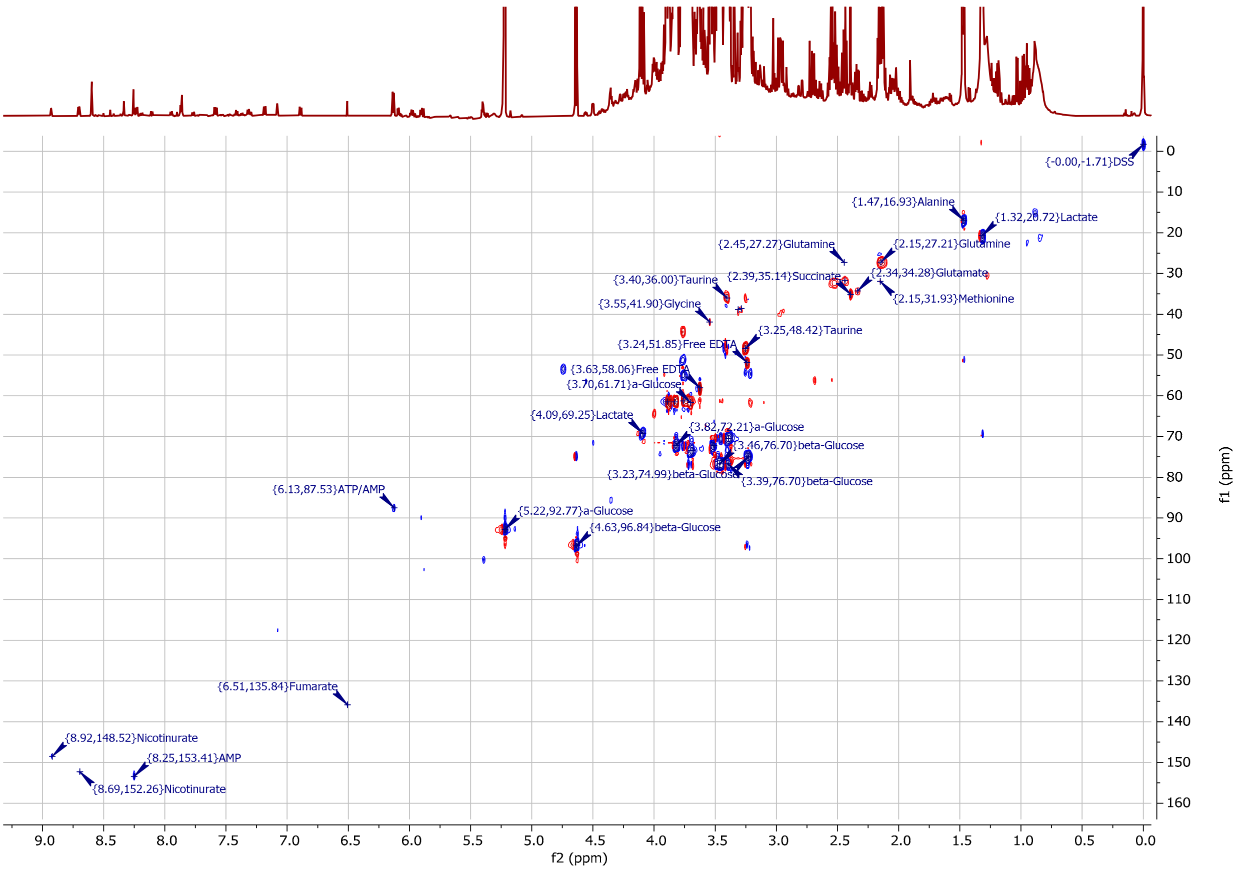

Figure S5. A fraction of HSQC spectrum for the liver sample (CKD 1 liver - aqueous phase) showing few metabolites present in higher concentration. CH3 and CH contours are represented by blue color and CH2 contours are represented by red color.

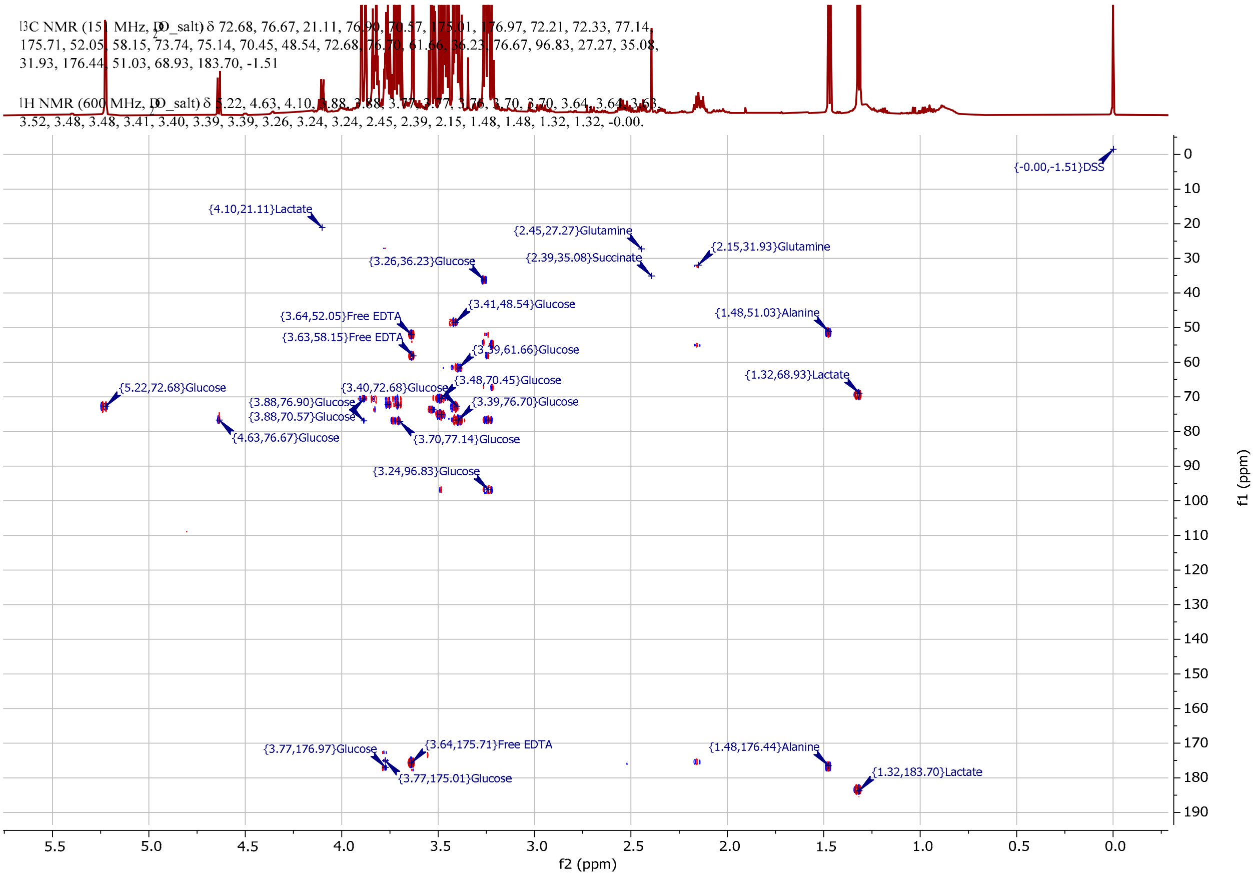

Figure S6. A fraction of HMBC spectrum for the liver sample (CKD 1 liver - aqueous phase) showing few metabolites present in higher concentration.
